# A 12-year follow up of principles learned in a pediatric dentistry Master of Public Health dual degree program applied to professional practice

**DOI:** 10.3389/fmed.2024.1322759

**Published:** 2024-04-24

**Authors:** Francisco Ramos-Gomez, Janni J. Kinsler, Stephanie Parkinson, Yan Wang

**Affiliations:** ^1^Division of Growth & Development, Section of Pediatric Dentistry, University of California, Los Angeles (UCLA) School of Dentistry, Los Angeles, CA, United States; ^2^Division of Oral and Systemic Health Sciences, Section of Public and Population Health, University of California, Los Angeles (UCLA) School of Dentistry, Los Angeles, CA, United States

**Keywords:** dental public health, oral disease prevention, dental health promotion, oral health disparities, social determinants of health, early childhood caries, underserved populations

## Abstract

**Introduction:**

Dental public health professionals play a critical role in preventing and controlling oral diseases. The purpose of this study was to assess the application of public health principles learned in a pediatric dentistry Master of Public Health (MPH) dual degree program to professional practice upon graduation.

**Methods:**

Semi-structured interviews were conducted with pediatric dentistry/MPH dual degree alumni who graduated from the program between 2012 and 2023. Interview questions inquired about characteristics of patient population, location of providers’ clinic/organization, whether the program was worthwhile to their practice and application of principles learned in the program to their professional practice.

**Results:**

Twenty of the 22 program alumni agreed to be interviewed. All alumni thought the program was extremely worthwhile to their practice. They felt the MPH component of the program gave them the public health background and tools they needed to provide comprehensive and holistic care to their patients. Additionally, all alumni reported applying the public health principles they learned in the program to their professional practice through leadership roles, research and teaching that focuses on oral disease prevention and the promotion of dental health.

**Discussion:**

Given the importance of a dental public health professionals’ role in reducing oral health disparities at the population level, more pediatric dentistry MPH dual degree programs are urgently needed. Additionally, more research is necessary to demonstrate the effectiveness of these programs, which will be critical to helping ensure the value of a dual degree in dentistry and public health is recognized and promoted worldwide.

## 1 Introduction

Early childhood caries (ECC) remains the most common chronic childhood disease in the United States, affecting 60–90% of school-age children (1, 2). Significant oral health disparities still exist in the burden of ECC (2, 3), with race/ethnicity and socioeconomic status acting as the most influential factors to these inequities (2–6). Among children 5 years of age or younger, ECC is most prevalent among American-Indian/Alaska Native individuals (71.3%) and Mexican-American individuals (41.5%), followed by non-Hispanic Black individuals (30.3%) and non-Hispanic White individuals (24.9%) (2, 7). ECC is also higher among children 2–5 years of age from poor families (<100% federal poverty level) compared with children from non-poor families (200% federal poverty level) (2). In 2008, financial struggles hindered 4.6 million children from receiving necessary dental care (8). These current disparities in children’s oral health represent a public health crisis (9).

In light of this, equipping future oral health professionals to address disparities is of the utmost importance, yet very few current dental education programs are devoted to training students and residents in prevention and public health (10). While a majority of dental schools heavily emphasize diagnosis, treatment, and restoration of individual patients in their training programs, they neglect to educate future oral health professionals on public health-related aspects of dentistry (11). Consequently, although graduates may enter the workforce well-equipped to care for the patients they see within their practice, many lack knowledge of oral health as it impacts the public as a whole, a sense of responsibility to the community they serve and a sufficient understanding of important topics relevant to public health including preventive care, disease management and oral health education (11, 12). This highlights a critical limitation in current dental education trends and the reinforces the need to reframe dental education in the context of public health. While lack of awareness of public health and its relevance to the field of dentistry is likely to hinder even the most skilled dentist from effectively promoting dental health in his or her community (11), a better understanding of public health for dental professionals has the potential to benefit the general population by improving oral health service and equipping dental providers to be promoters of holistic health (11).

As of 2019, there were 29 dual degree programs in the United States and Canada that offered a Master of Public Health (MPH) along with a dental degree (13), and as of 2020 15 Dental Public Health (DPH) residency programs had been accredited by the Commission on Dental Accreditation (CODA) (14). DPH is a specialty recognized by the American Dental Association (ADA) which supports their vision and mission of improving oral health for the population at large (15). Most of the DPH programs require having an MPH degree or its equivalent; however, there are a few dual-degree programs that offer MPH degrees as part of their DPH programs. A couple of studies have explored the challenges and opportunities DPH residents and graduates face during and after their training (15), perspectives on teaching methods of their DPH program and application of DPH competencies (16). These studies found that DPH graduates faced challenges finding DPH-related jobs post-graduation in addition to educational and financial challenges (15) and that more time should be dedicated to certain public health-related competencies including leadership and policy and advocacy (16).

We found just one study evaluating the impact of completing an MPH degree during dental school on general dentists’ practice behaviors, volunteerism and attitudes towards dental education, practice preparedness and the dental profession (17). This study found that dentists with an MPH were more likely to practice in a public health setting than those without an MPH degree (17). There is currently a lack of literature on pediatric dentistry MPH dual degree programs. Given the high prevalence of ECC (1, 2) and the significant oral health disparities associated with ECC (2, 3), training pediatric dentists in the principles of public health is crucial to preventing and controlling ECC in infants and children at the population level. The UCLA School of Dentistry’s Section of Pediatric Dentistry has an innovative pediatric dentistry/MPH dual degree program that focuses on framing oral health in the context of public health to train the next generation of pediatric dentists to make a difference beyond the traditional dental office (11, 18). To enhance dental public health training for pediatric dentistry residents, a collaboration was developed with the UCLA Fielding School of Public Health through their executive MPH for Community Health Sciences program. UCLA’s pediatric dentistry/MPH dual degree program financially supports selected residents in earning their MPH while they complete their pediatric dental residency. The overall goal of UCLA’s program is to enhance the pediatric dental residency program with a public health focus to best equip residents to address the social determinants of health and public health consequences of oral health diseases. Since the inception of the program in 2010, 22 residents have earned their pediatric dentistry/MPH dual degree.

The purpose of this longitudinal panel design follow-up study was to evaluate the application of public health principles learned in the UCLA’s School of Dentistry’s pediatric dentistry/MPH dual degree program to their professional practice upon graduation.

## 2 Methods

### 2.1 Sample and recruitment

The UCLA School of Dentistry’s pediatric residency program recruits 1–2 applicants each academic year to participate in the pediatric dentistry/MPH dual degree program. The executive MPH program is funded through a grant from the Health Resources and Services Administration (HRSA). All 22 residents who graduated from the program between 2012 and Spring of 2023 were sent an e-mail to participate in a semi-structured interview. Residents who graduated the program between 2012 and 2019 were interviewed in Spring 2020. For residents graduating between 2020 and Spring of 2023, interviews were conducted 6 months post-graduation.

This study met the criteria for an exemption by the UCLA Institutional Review Board (IRB; # 16–000185).

### 2.2 Curriculum for pediatric dentistry/MPH dual degree residents

The pediatric dentistry/MPH dual degree program takes 2 years to complete. The pediatric dentistry component of the curriculum includes the following nine evidence-based training modules designed to align with the Commission on Dental Accreditation (CODA) Standards for advanced education in pediatric dentistry: (1) disease prevention, management and risk assessment; (2) ethics and professionalism, (3) cultural competency; (4) applied statistics and research methods; (5) community partners; (6) interprofessional education/training; (7) quality improvement; (8) policy and advocacy; and (9) special needs children. These nine modules supplement the existing pediatric dental residency clinical curriculum. A detailed description of the training modules is described in previous publications (19–21).

The 2 year executive MPH curriculum, which requires residents to attend classes one weekend per month, includes the following courses: program planning, research and evaluation; community health sciences; introduction to health policy and management; principles of epidemiology; introduction to biostatistics; introduction to environmental health; research in community and patient health education; health promotion and education; social marketing for health promotion and communication; politics of health policy; information technology for health promotion and communication; and community organization for health (see Table 1 for a detailed description of the courses). Given the public health consequences of oral health disease and growing oral health disparities among children from underserved and vulnerable populations (9, 22), viewing oral health in the context of a public health framework is an important aspect of the programs’ multidisciplinary approach to training dental residents to have an impact beyond the traditional dental office setting (12, 18). All pediatric dentistry/MPH dual degree residents are required to complete an MPH project that is pediatric oral health related. The MPH project gives students an opportunity to apply knowledge and skills gained through coursework to a specific problem of significance in oral health education and health promotion.

**Table 1:** Description of MPH courses.

Program planning, research, and evaluation in community health sciences	Development, planning, and administration of public health programs in community settings. Introduction to a range of research methods and techniques used in designing and conducting health research, with particular emphasis on evaluation of community-based public health programs
Community health sciences	Basic concepts, relationships, and policy issues in field of community health, variability in definitions of health and illness, correlates of health and illness behavior, impact of social and community structure on health status, major contemporary approaches to health promotion and health education at community level. Use of comparative international perspective
Introduction to health policy and management	Structure and function of the U.S. health care system, health care policy, and issues and forces shaping its future.
Principles of epidemiology	Introductory course to provide students with a broad and comprehensive overview of concepts of epidemiology including evaluating public health problems in terms of magnitude, person, time and place; critiquing epidemiologic studies; identifying and accessing key sources of data for epidemiologic assessment; using epidemiologic methods and calculating basic epidemiology measures for operational purposes; and communicating basic principles of epidemiology such as definitions of populations, sources of bias, causation for morbidity and mortality, risk and protective factors, and basics of study design
Introduction to biostatistics	Introduction to methods and concepts of statistical analysis. Sampling situations, with special attention to those occurring in biological sciences. Topics include distributions, tests of hypotheses, estimation, types of error, significance and confidence levels, sample size
Introduction to environmental health	Introduction to environmental health, including coverage of sanitary principles and chronic and acute health effects of environmental contaminants
Research in community and patient health education	Application of conceptual, theoretical, and evaluation skills to community-based health education risk-reduction programs. Computer applications, data management, and research methodologies taught through microcomputer and mainframe computer management and analysis of program databases
Social marketing for health promotion and communication	Planning, creating, implementation, and evaluation of comprehensive health communication campaigns, including use of social marketing practices and strategies of audience research, marketing psychology, creative message development, branding, comprehensive media use for dissemination, transmedia
Politics of health policy	Examination of politics of health policy process, including effects of political structure and institutions; economic and social factors; interest groups, classes, and social movements; media and public opinion; and other factors
Information technology for health promotion and communication	Health literacy, Internet use and health communication, design of health communication materials using digital media that integrates practice and theory and includes websites, print materials, short videos, curricula, and training materials. Laboratory sessions for materials production
Community organization for health	Theory and practice of community organizations, including models and strategies of community organization and their application to health problems and health policy. Particular attention to use of community organization for health promotion and to change public policy

### 2.3 Data collection

The semi-structured interview guide included the following six questions: (1) Please describe your current practice in terms of location, type of practice (private/public, Federally Qualified Health Center [FQHC]), type of population served (e.g., low-income/Medi-Cal patients, race/ethnicity of patient population, special needs populations, insurance coverage); (2) Was the pediatric dentistry/MPH dual degree program worthwhile to your professional practice (Probes: Why? Why not? Please provide some examples); (3) Do you apply the public health concepts learned in the program to your current practice (Probes: If yes, how? if not, why not?); (4) Are you currently using your pediatric dentistry/MPH dual degree in any of the following capacities: teaching, research, assume a public health-related leadership role in an organization? (Probe: if yes, please provide examples); and (5) Do you have any suggestions for improving the pediatric dentistry/MPH dual degree program? The interview guide was developed by the project investigators. Questions were based on the core competencies covered during the pediatric dentistry/MPH dual degree program. Interviews were conducted by a professional qualitative interviewer via telephone and were digitally recorded. Interviews lasted approximately 20–30 min.

### 2.4 Data analysis

Interviews were professionally transcribed and analyzed by the project evaluator using Atlas.ti 22 (Scientific Software Development GmbH, Berlin, Germany). Each transcript was read in its entirety. After reading each transcript a second time, representative codes to emergent themes were identified and assigned. Although some codes were identified *a priori* based on predefined questions in the interview guide, other codes were created as new themes emerged. Text passages in the transcripts were read multiple times by the project evaluator and program manager, and coding was refined during this iterative analytic process. Next, text with the same code for all interviews was extracted and formatted into tables highlighting the major themes and supporting quotes. Any discrepancies were discussed and resolved by the project evaluator and program manager. Finally, specific quotations were chosen to represent the emergent themes.

## 3 Results

### 3.1 Alumni demographics

A total of 20 interviews were conducted with alumni who graduated from the UCLA School of Dentistry’s pediatric dentistry/MPH dual degree program between 2012 and Spring 2023.

Alumni demographics are presented in Table 2. Over half (55%) of alumni entered the pediatric dentistry/MPH dual degree program when they were between 20 and 29 years of age. Approximately two-thirds (65%) of alumni were female. Over one-third (35%) of alumni were Hispanic/Latino individuals followed by 25% who were Asian individuals and 20% each for White/Caucasian individuals and Black/African American individuals. Almost half (45%) identified as an underrepresented minority.

**Table 2:** Demographics of the pediatric dentistry/MPH dual degree residents.

Demographics	*N* (%)
Age when resident entered the pediatric dentistry/MPH dual degree program	
20–29	11 (55%)
30–39	7 (35%)
40–49	2 (10%)
Gender
Female	13 (65%)
Male	7 (35%)
Race/ethnicity	
White/Caucasian individuals	4 (20%)
Black/African American individuals	4 (20%)
Hispanic/Latino individuals	7 (35%)
Asian individuals	5 (25%)
Self-reported rural background	
Yes	3 (15%)
No	17 (85%)
Self-reported underrepresented minority	
Yes	9 (45%)
No	11 (55%)

### 3.2 Characteristics of alumni’s patient population and professional practice

Most of the pediatric dentistry/MPH alumni work at more than one clinic (a combination of public and private) serving a mix of low- and high-income White individuals, Hispanic/Latino individuals, African American individuals and Asian individuals. A few alumni work at FQHCs. Six alumni practice in a dental Health Professional Shortage Area (HPSA), 8 work in a Medically Underserved Area (MUA) and 3 work in both an HPSA and MUA areas (Table 3). Most of the clinics that alumni work at accept public health insurance (e.g., Medi-Cal, Medicaid, care credit). All alumni reported seeing special needs patients (e.g., patients with autism, facial deformities, Downs Syndrome, other major syndromes, and patients on organ transplant lists).

**Table 3:** Location of alumni’s current practice by zip code, city, Health (dental) Professional Shortage Area (HPSA) and Medically Underserved Area (MUA).

Zip code	City	Dental HPSA	MUA
CA 91723	Covina	No	No
NY 10037	New York	Yes	Yes
CA 93035	Oxnard	Yes	No
CA 95032	Los Gatos	No	No
CA 94954	Petaluma	No	Yes
MI 48180	Taylor	No	No
CA 90749	Carson	No	No
CA 93551	Palmdale	Yes	No
CA 92868	Orange	No	No
CA 90089	Los Angeles	Yes	Yes
CA 92071	Santee	No	No
CA 94303	East Palo Alto	No	Yes
CA 90808	Long Beach	No	No
CA 90740	Seal Beach	No	No
CA 93003	Ventura	No	No
CA 93111	Santa Barbara	No	Yes
CA 90057	Los Angeles	No	Yes
CA 90057	Los Angeles	No	Yes
CA 93306	Bakersfield	Yes	No
GA 31204	Macon	Yes	Yes

Many alumni currently teach in a university setting. A few are full or part-time faculty at schools of dentistry, and one alumnus is Chair of Pediatric Dentistry at a university. Two alumni hold a public health-related leadership role in an organization serving vulnerable populations. A few alumni reported teaching residents or pre-doctoral students as part of their current professional practice. For example, one alumnus works with dental residents on their research projects by helping them design their research proposals, conduct analyses and write manuscripts for publication. One alumnus consults for a dental care-related philanthropic organization and teaches seminars on the basics of dental anatomy, dental practice, treatment planning and patient management. Almost all alumni reported doing oral health outreach to underserved communities. Many reported going to local schools, libraries, Boys & Girls Clubs of America, Head Start programs and other community-based organizations to educate them about dental health. While all alumni reported conducting research while they were in the program, a few continue to be involved in research. For example, one alumnus was funded to assess the oral health status of children under the age of 18 in refugee camps in Jordan and continues to conduct research on acculturation and utilization of oral health care services in New York. A few other alumni reported conducting quality assurance-related research as part of their job responsibility in their current dental practice.

The following two major themes emerged from the 20 interviews: (1) the pediatric dentistry/MPH dual degree program has been worthwhile to alumni’s professional practice and (2) alumni are applying content learned in the pediatric dentistry/MPH dual degree program to their current practice. Table 4 presents supporting quotes for each of the two themes.

**Table 4:** Pediatric dentistry/MPH alumni interview themes and supporting quotes.

Themes	Supporting quotes
Pediatric dentistry/MPH dual degree program has been worthwhile to alumni’s professional practice	“The MPH has allowed me to really consider a lot more than just treating the tooth and cavity and to look at the bigger picture of why things are happening and how to be effective in approaches towards making things different” “I think it’s definitely been worthwhile. I have always wanted to get more understanding public health background of things, and so having this dual program has really been a valuable impact on the care I’m able to provide” “I will be working at a federally qualified health center. So, that’s another reason why I wanted to get the MPH. Having all the background knowledge I felt was necessary to provide the best care for my patients, poor populations and underserved populations and patients that have special healthcare needs. I’m committed to serving in this population” “I think some of the biggest strengths of the MPH is us learning how to do a program planning and evaluation, which is something that we very thoroughly learned” “There’s a lot of other things that we were also taught, in terms of policy and advocacy, in terms of environment regulations, in terms of community organizing and also our basic science courses like biostats, epidemiology, all of that good stuff, good foundation” “The MPH program gave me a new perspective- more population-based and awareness regarding the needs of underserved populations and lack of access to care - regarding providing care to my patients and their families”
Alumni are applying content learned in the pediatric dentistry/MPH dual degree program to their current practice	“Some of the things I incorporated [in my current practice] was through my research. It had to do with whether the caries risk assessment and its role in caries experience with patients. I found that the counseling made an impact on the caries experience index (better oral health with patients who got caries risk assessment counseling vs. those that did not). I found out those who got risk assessment counseling had better oral health. Motivated me to continue using the caries risk assessment counseling” “The research methods class was very helpful in understanding how to read a published literature and distinguish what is important and what not to hone in. So, during our lit reviews on Mondays, I was able to use that skill from that research class and kind of analyze the literature a little bit better to know better materials and practice” “It’s the thought process of public health that has had the biggest impact in my practice (e.g., learning how to make an impact in controlling oral health disease on a larger scale, how to create programs for oral health disease, and how to work effectively in a group setting)” “The dental practice I work at is in the process of opening more offices, so I have been able to apply what I learned in program planning and business management/accounting/financing courses to my current responsibilities of helping the practice expand. These courses were very helpful since I wasn’t exposed to them in dental school.” “Program development (e.g., whether programs at clinic are running efficiently), systems management (where to put resources, how referrals and data are logged into the computer, logistics), quality assessment, policy (changes in reimbursement, understanding new policies as they relate to her practice and patients) have been most useful to me” “I apply public health concepts in work and in my research every day. I am currently looking at the relationship between acculturation and utilization of oral health practices in New York. Previously, I conducted research in Jordan where I assessed the oral health status of children under the age of 18 in refugee camps” “The biostatistics course has been useful for understanding and conducting research and the program planning courses have been useful for teaching us how to assess the needs of underserved populations and how to reach out to communities in need” “The MPH program provided me with a well-rounded education and has allowed me to look at the relationship between medical providers and dental providers through a different lens. Many of the medical care providers I interact with in my dental practice have also received an MPH, so we have a common language and better understand each other. This commonality helps in providing comprehensive care to our patients” “Absolutely. Within my practice, we have quality metrics that we need to meet, so understanding what goes into those metrics, why those metrics are chosen, if those metrics are good helps not only give context to why we are doing what we are doing and how we are doing what we are doing, but also whether we need to reevaluate those measures to begin with. That’s how the MPH gave us a context to these overall strategies that the organization uses”

### 3.3 Pediatric dentistry/MPH dual degree program has been worthwhile to alumni’s professional practice

All alumni thought the pediatric dentistry/MPH dual degree program was extremely worthwhile to their professional practice. They felt the MPH program provided them with the public health background and tools they needed to provide comprehensive and holistic care to their patients. According to one alumnus, the *“MPH has allowed me to really consider a lot more than just treating the tooth and cavity and to look at the bigger picture of why things are happening.”* A few alumni reported being passionate about giving back to underprivileged minorities, wanting to make a difference in the field of pediatric dentistry through focusing on prevention and feeling the public health skills learned in the MPH program have given them that opportunity. Several alumni mentioned the program planning and evaluation course as a major strength of the program. One alumnus mentioned that *“without that degree, I do not know if I would have felt qualified or comfortable taking the position I am in now because I have the perspective of program planning, how to write grants and how to collaborate with multiple researchers for projects to get health initiatives funded at the local, state and federal levels.”*

### 3.4 Alumni are applying content learned in the pediatric dentistry/MPH dual degree program to their current practice

Several alumni reported applying the public health principles they learned in the program planning, research and evaluation course to their professional practice. A couple of alumni were involved in developing, implementing and evaluating prevention-focused dental programs at their clinic, and a few other alumni were involved in systems-based evaluation. For example, one alumnus was evaluating whether clinic programs were running efficiently and was also reevaluating whether the current metrics being used to assess quality assurance were the best measures available. According to this alumnus, *“one of the interim site directors came to me and said if you think some of these measures should be changed or we need additional measures, we are open to hearing that.”* A few alumni were applying what they learned in the health policy and management course. One alumnus mentioned that *“health policy and management helped me understand how dental services are covered and reimbursed and now I can help my patients who have issues paying for certain treatments.”* Alumni are also applying what they learned in the biostatistics course and their community-based education and communication courses. For example, one alumnus described using “*pictographic*” tools to educate non-English speaking patients about proper oral health care. Another alumnus used principles from the community organization for health and social marketing for health promotion and communication courses to assess dental needs in underserved communities and then developed an oral health promotion social marketing program for caries prevention.

## 4 Discussion

To our knowledge, this is the first longitudinal panel design follow-up study assessing how practitioners are applying the public health principles they learned in a pediatric dentistry/MPH dual degree program to their professional practice. This study demonstrated that all alumni were advocating for increasing access to affordable oral health care services for underserved and special needs populations with a focus on community-based oral disease prevention and dental health promotion in their current practice. Almost three-quarters of alumni reported practicing in a HPSA or MUA-designated clinic. Specifically, two alumni reported holding public health/pediatric director leadership positions in FQHCs, several were directly involved in population-based research studies that focused on increasing access to and utilization of oral health care services and patient quality of care, and a few were teaching public health courses to pre- and post-doctoral providers as part of their job responsibilities. These findings are promising and lend support to the positive impact an MPH degree can make among pediatric dental providers.

All alumni thought the dual degree program was extremely worthwhile to their professional practice and felt the MPH degree provided them with the public health background and tools they needed to provide comprehensive and holistic care to their patients. Alumni reported applying several of the public health principles they learned in the program to their professional practice. The program planning, research and evaluation, health policy and management and community organization for health courses were mentioned as being particularly useful to the alumni’s professional practice. The dual degree residents in the program are required to complete an MPH project based on the core competencies covered in the program and they learn how to do this in their program planning, research and evaluation course. For example, a few alumni developed, implemented, and evaluated a community oral health worker “Promotoras” program for Early Head Start (23, 24). Other alumni have worked on large nationwide databases to examine the relationship between social determinants of health (SDOH) and prevalence and incidence of dental caries and other oral diseases (25–27). Being able to influence SDOH is an important part of a DPH professional’s role as it recognizes the effect that adverse socio-economic and environmental factors can have on poor oral health outcomes (28). All alumni thought the MPH project provided them with the skills needed to address real-world problems once they graduated from the program, and many alumni reported being involved in public health-related research in their current practice.

A few alumni reporting applying what they learned in the health policy and management course to their professional practice. For example, alumni mentioned the information they learned in the course provided them with a better understanding of how dental services are covered and reimbursed which has translated into them being able to help their patients who have issues paying for certain treatments, and taught them about business management, which one alumnus has found extremely helpful with opening and managing new dental clinics. Every year, in collaboration with the American Academy of Pediatric Dentistry (AAPD), the UCLA program brings the pediatric dentistry/MPH dual degree residents to Washington, DC where they attend meetings with legislators to promote greater access to high quality oral health care, especially among vulnerable populations, as well as “mandatory” age-one dental care visits (a crucial first step in the prevention of ECC) (2, 29). This opportunity provides the residents a great way to network with their peers, university faculty and politicians at the state and national levels.

Several alumni reported applying what they learned in the community-based courses to their current practice. UCLA’s relationships with other community-based organizations, such as Early Head Start, Head Start and Women Infants & Children, provide residents with opportunities to work at and network with underserved organizations, which is a key focus of the pediatric dentistry/MPH dual degree program. Networking with community-based organizations is enhanced through UCLA’s Infant Oral Care Program (IOCP), which is located at a local community health clinic in Los Angeles and provides culturally sensitive preventive oral health for children 0–5 years of age (19). An IOCP rotation is mandatory for all UCLA dental residents, including the pediatric dentistry/MPH dual degree residents (19).

Comparing our findings with other studies is challenging as there are currently no other published studies that have evaluated the impact of a pediatric dentistry/MPH dual degree program on graduates’ professional practice. However, a study which evaluated the impact of completing an MPH degree during dental school on general dentists’ practice behaviors found that alumni who obtained an MPH degree were more likely to practice in a public health context compared with those who did not obtain an MPH degree (17). While these findings lend support to our results, the study did not examine how graduates were implementing the public health principles they learned in the program to their current practice.

There were limitations to our study. Although the strength of qualitative data is to gather detailed information on a topic that cannot always be obtained using quantitative methods, a limitation of qualitative studies is lack of generalizability (30). Our study was conducted at one university in the US and cannot be generalized to other pediatric dentistry/MPH programs. Small sample size is another limitation of qualitative studies, although it is common for samples to be small when conducting in-depth interviews because the purpose is to generate information by deeply exploring a topic or issue among a specific target population (30, 31).

In conclusion, our findings demonstrate that graduates of a pediatric dentistry/MPH dual degree program were applying the public health principles they learned in the program to their professional practice through leadership roles, research and teaching that focused on community-based oral disease prevention and the promotion of dental health. Given the importance of viewing oral health in the context of a public health framework, the pediatric dentistry/MPH dual degree program is an important aspect of the UCLA School of Dentistry’s multidisciplinary approach to training pediatric dental residents to have an impact beyond the traditional dental office setting. More pediatric /MPH dual degree programs are urgently needed to affect oral health of children and their families at the population level, and research showing the effectiveness of these programs will be critical to helping ensure the value of a dentistry/MPH dual degree is recognized and promoted worldwide (28).

## Data availability statement

The raw data supporting the conclusions of this article will be made available by the authors, without undue reservation.

## Ethics statement

The studies involving humans were approved by UCLA Institutional Review Board (IRB; # 16-000185). The studies were conducted in accordance with the local legislation and institutional requirements. The ethics committee/institutional review board waived the requirement of written informed consent for participation from the participants or the participants’ legal guardians/next of kin because it was exempt because the participants were health professionals who were graduates of UCLA's pediatric dentistry MPH dual degree program.

## Author contributions

FR-G: Funding acquisition, Investigation, Supervision, Writing – review & editing. JK: Conceptualization, Data curation, Formal analysis, Methodology, Writing – original draft. SP: Conceptualization, Project administration, Validation, Writing – original draft. YW: Methodology, Validation, Writing – review & editing.
